# Osteocyte regulation of orthodontic force-mediated tooth movement *via* RANKL expression

**DOI:** 10.1038/s41598-017-09326-7

**Published:** 2017-08-18

**Authors:** Ayumi Shoji-Matsunaga, Takehito Ono, Mikihito Hayashi, Hiroshi Takayanagi, Keiji Moriyama, Tomoki Nakashima

**Affiliations:** 1 Department of Cell Signaling, Graduate School of Medical and Dental Sciences, Tokyo Medical and Dental University (TMDU), Yushima 1-5-45, Bunkyo-ku, Tokyo 113-8549 Japan; 2 Department of Maxillofacial Orthognathics, Graduate School of Medical and Dental Sciences, Tokyo Medical and Dental University (TMDU), Yushima 1-5-45, Bunkyo-ku, Tokyo 113-8510 Japan; 3 Department of Immunology, Graduate School of Medicine and Faculty of Medicine, The University of Tokyo, Hongo 7-3-1, Bunkyo-ku, Tokyo 113-0033 Japan; 4 Precursory Research for Embryonic Science and Technology (PRESTO), Japan Science and Technology Agency (JST), Yushima 1-5-45, Bunkyo-ku, Tokyo 113-8549 Japan; 5 Core Research for Evolutional Science and Technology (CREST), Japan Agency for Medical Research and Development (AMED), Yushima 1-5-45, Bunkyo-ku, Tokyo 113-8549 Japan

## Abstract

Orthodontic tooth movement is achieved by the remodeling of the alveolar bone surrounding roots of teeth. Upon the application of orthodontic force, osteoclastic bone resorption occurs on the compression side of alveolar bone, towards which the teeth are driven. However, the molecular basis for the regulatory mechanisms underlying alveolar bone remodeling has not been sufficiently elucidated. Osteoclastogenesis is regulated by receptor activator of nuclear factor-κB ligand (RANKL), which is postulated to be expressed by the cells surrounding the tooth roots. Here, we show that osteocytes are the critical source of RANKL in alveolar bone remodeling during orthodontic tooth movement. Using a newly established method for the isolation of periodontal tissue component cells from alveolar bone, we found that osteocytes expressed a much higher amount of RANKL than other cells did in periodontal tissue. The critical role of osteocyte-derived RANKL was confirmed by the reduction of orthodontic tooth movement in mice specifically lacking RANKL in osteocytes. Thus, we provide *in vivo* evidence for the key role of osteocyte-derived RANKL in alveolar bone remodeling, establishing a molecular basis for orthodontic force-mediated bone resorption.

## Introduction

Bone is constantly being renewed by the balanced activities of osteoblastic bone formation and osteoclastic bone resorption, both of which occur at the bone surface. This restructuring process, called “bone remodeling”, is crucial for maintaining normal bone mass, architecture, strength and mineral homeostasis^1–3^. Bone remodeling is stringently regulated by the communication that takes place between bone component cells; osteoclasts, osteoblasts and osteocytes^4, 5^. During the course of bone remodeling, the bone resorption performed by osteoclasts precedes the bone formation performed by osteoblasts. Based on the fact that osteocytes are embedded in the bone matrix and these cells extend dendrites that communicate with adjacent cells, it has been proposed that osteocytes control bone remodeling by sensing mechanical stimuli^5–7^. In response to mechanical loading, bone morphology is modified by coordinated bone remodeling such that it adapts to the loading condition.

Alveolar bone, which supports teeth, is also renewed throughout the human lifespan. The mechanical environment generated by the oral and maxillofacial tissues around the teeth regulates alveolar bone remodeling^8, 9^. In orthodontic treatment, orthodontic devices are applied to modify the mechanical environment in maxillofacial structures so as to obtain the optimal occlusion in functional harmony as well as well-balanced dental and facial esthetics in each individual. In the course of orthodontic tooth movement, the alveolar bone surrounding the tooth roots undergoes well-organized bone remodeling^8, 9^. Upon the application of orthodontic force, the mechanical force stimulates the cells within the periodontal tissue (mainly the periodontal ligament and alveolar bone), leading to cellular responses that result in alveolar bone remodeling. Bone resorption occurs at the compression side of the alveolar bone, towards which the teeth are driven, whereas bone formation is induced in the opposite side (the tension side)^8–10^.

Osteoclasts are cells of hematopoietic origin that decalcify and degrade the bone matrix by hydrochloric acid and proteolysis, respectively^1, 2, 4^. These cells are large multinucleated cells formed by the fusion of precursor cells of monocyte/macrophage lineage. The differentiation of osteoclasts is supported by mesenchymal linage cells, a process which is mediated by receptor activator of nuclear factor κ-B ligand (RANKL; encoded by the *Tnfsf11* gene)^1–3^. RANKL is essential for osteoclastogenesis because mice lacking the *Tnfsf11* gene exhibit severe osteopetrosis accompanied by a defect in tooth eruption owing to a lack of osteoclasts^11^. Osteoblasts and bone marrow stromal cells were previously thought to be the major cell types that express RANKL to support osteoclastogenesis^12, 13^. However, it has been clearly demonstrated using a conditional knockout system that the RANKL derived from osteocytes contributes more significantly to bone remodeling, rather than that from osteoblasts^14–17^.

Osteocytes, the most numerous cells in bone, are stellate-shaped cells enclosed within a bone lacuno-canalicular network and have been shown to function as mechanosensory cells in bone^4, 5^. Although recent reports suggest that osteocytes are involved in tooth movement in response to orthodontic force^18, 19^, the underlying regulation mechanism for the effect remains to be elucidated. Here we show that osteocytes substantially express RANKL among the periodontal tissue component cells, and demonstrate that the osteocyte derived-RANKL regulates osteoclastogenesis during orthodontic tooth movement. This study establishes the crucial role of osteocyte RANKL-mediated osteoclastogenesis in alveolar bone remodeling, which can facilitate the development of novel therapeutic strategies in orthodontics.

## Results

### RANKL mediates osteoclastogenesis by orthodontic force

**Figure 1:**
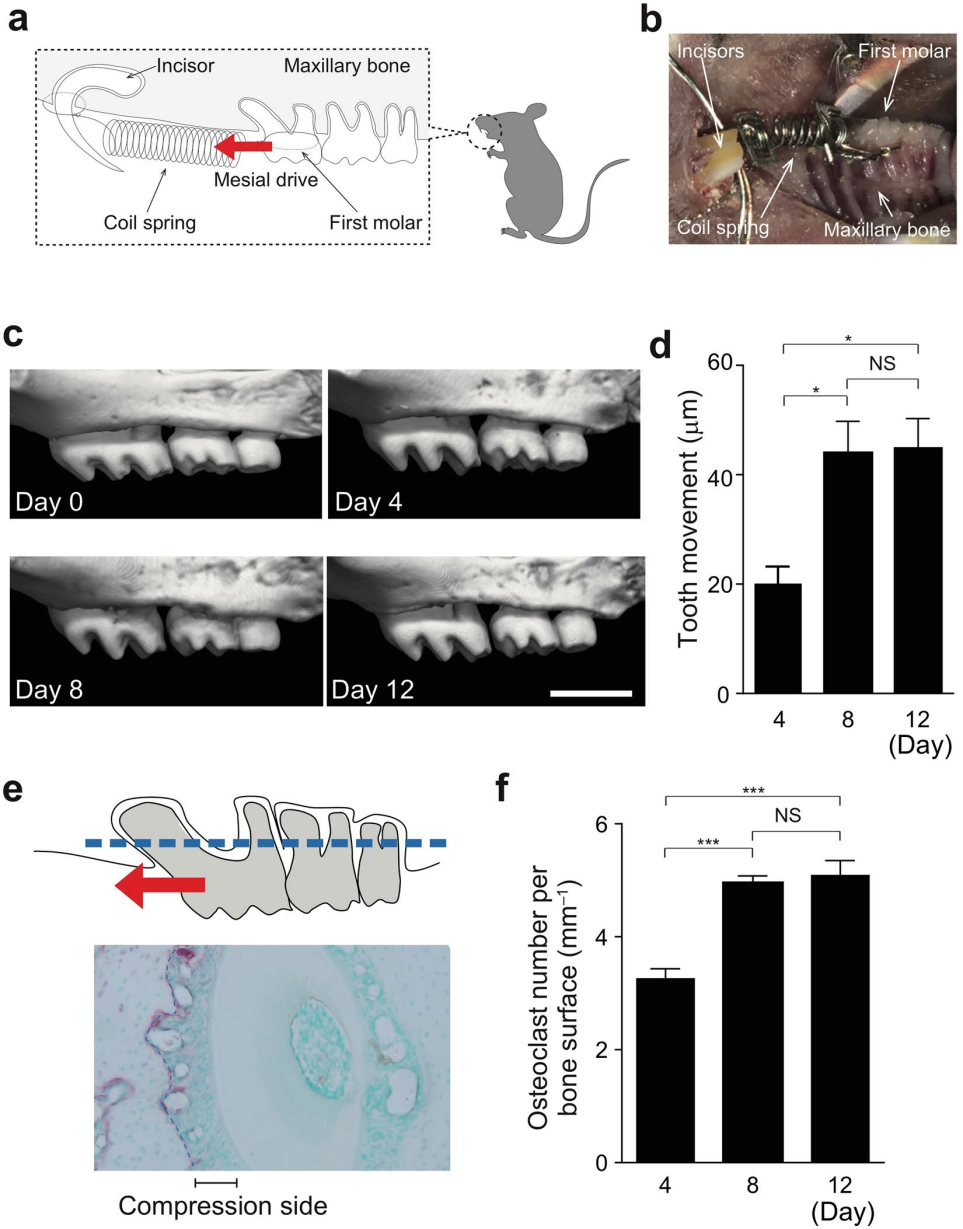
Experimental tooth movement model. (**a**) Schematic diagram of orthodontic tooth movement. The upper first molar was moved mesially by a closed–coil spring. The red arrow indicates the direction of orthodontic force. (**b**) Intra–oral photograph. (**c**) Tooth movement during the experiment time course. Representative micro-CT images in wild–type mice after spring insertion (0, 4, 8 and 12 days). (**d**) Quantification of tooth movement in each group (n = 3 per time point). (**e**) Measurement of osteoclasts on alveolar bone around the distal buccal root of the upper first molar (area enclosed by the dotted line). (**f**) The number of osteoclasts on the alveolar bone surface (n = 3 per time point). Scale bar: 1 mm. Error bars, means ± s.e.m.; *P < 0.05; ***P < 0.001; NS, not significant.

To mimic orthodontic tooth movement in patients, we utilized a mouse model in which the upper left first molars were driven mesially by nickel-titanium open coil springs inserted between these teeth and the alveolar bone around the incisors (Fig. 1a,b). We measured the orthodontic tooth movement using micro-CT, with which high-resolution images can be acquired, enabling precise analyses (Fig. 1c and Supplementary Fig. 1a). Tooth movement reached a maximum 8 days after spring insertion in this model (Fig. 1d). The number of osteoclasts dramatically increased on the compression side of the periodontal ligament space with its maximum on day 8 (Fig. 1e,f). The number of osteoblasts was also increased on the tension side and maximized on day 8 (Supplementary Fig. 1b). The following analyses were performed on day 8.

**Figure 2:**
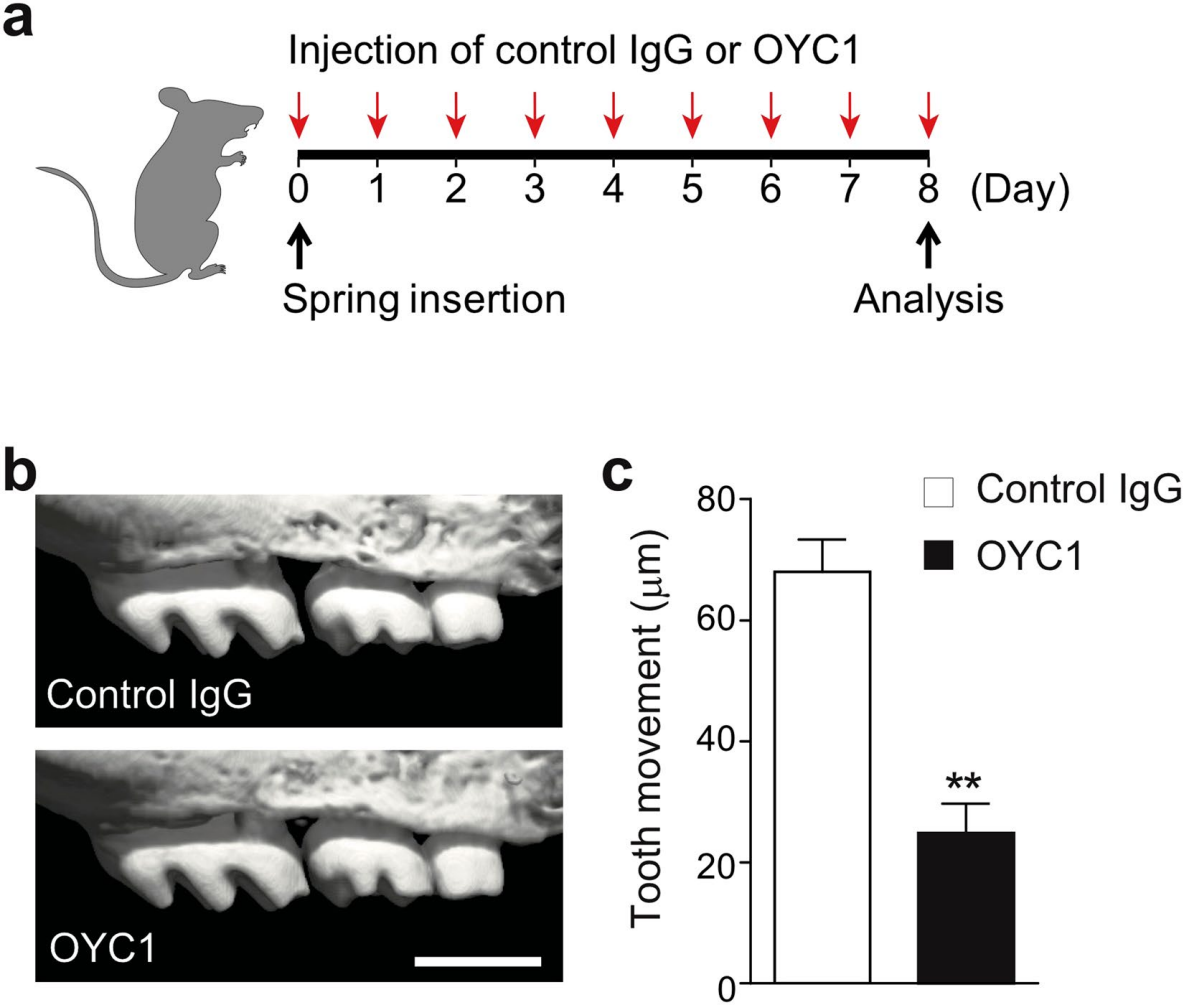
Effect of anti-mouse RANKL monoclonal antibody (OYC1) on tooth movement. (**a**) Schematic diagram of the injection time course. OYC1 or control IgG was injected locally into the buccal and palatal gingiva around the upper left first molar of wild-type mice. (**b**) Representative micro-CT images of a moved tooth after treatment of OYC1 or control IgG. (**c**) Quantification of tooth movement in each group (n = 3–4). Error bars, means ± s.e.m.; **P < 0.01.

Pharmaceutical inhibition of RANKL was performed to clarify the functional evidence of RANKL on orthodontic tooth movement. A neutralizing antibody against mouse RANKL (OYC1) was administered locally into the buccal and palatal gingiva around the upper left first molar in wild-type mice (Fig. 2a). Serial injection of OYC1 resulted in a significant reduction in orthodontic force-induced tooth movement, whereas isotype control antibody did not exert any such effect (Fig. 2b,c). These data show that RANKL plays an essential role in orthodontic tooth movement.

### Osteocytes are the major source of RANKL in periodontal tissue

**Figure 3:**
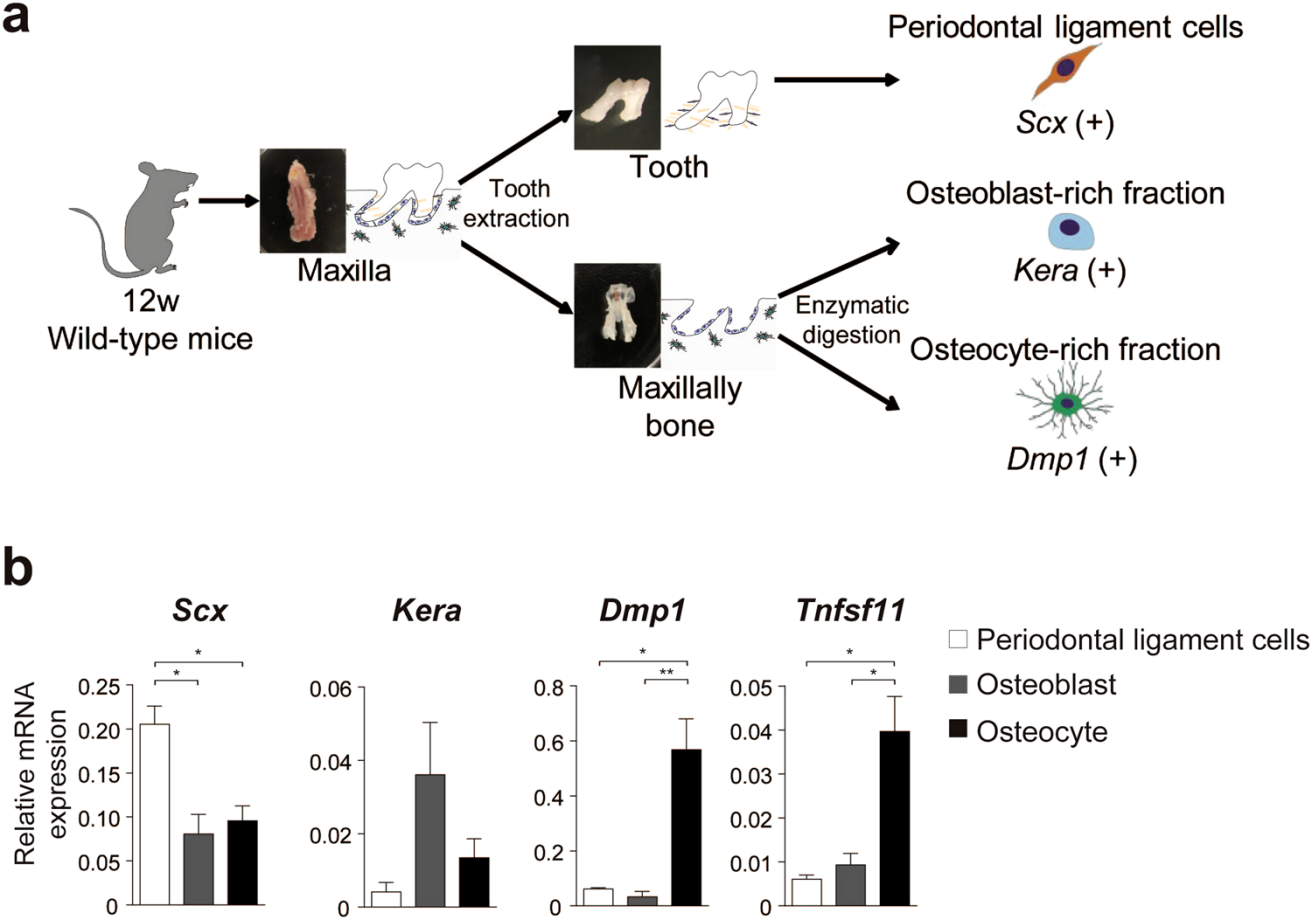
RANKL expression in periodontal tissues. (**a**) Establishment of isolation of cells from periodontal tissue. Periodontal ligament cells, osteoblasts and osteocytes were collected according to the schematic flow chart (see methods). (**b**) Profiling of gene expression in periodontal ligament cells, osteoblasts and osteocytes (quantitative RT-PCR analysis) (n = 5 per each fraction). Statistical analysis of *Dmp1* expression was carried out using Kruskal-Wallis test and Dunn’s multiple comparison test. Error bars, means ± s.e.m.; *P < 0.05; **P < 0.01.

In order to identify the relevant osteoclastogenesis–supporting cells among the periodontal component cells, we investigated the expression of *Tnfsf11* (encoding RANKL) in periodontal ligament cells, osteoblasts and osteocytes. We established a novel isolation method to obtain these cells from periodontal tissue (Fig. 3a). Molars were extracted from the maxillary bone, and periodontal ligament cells around the teeth roots were harvested. To fractionate the population of osteoblasts and osteocytes, the remaining alveolar bone was sequentially digested with collagenase/dispase (see methods).

To confirm the purity of the three obtained fractions (periodontal ligament cells, osteoblast-rich fraction and osteocyte-rich fraction), we examined the expression of marker genes of these fractions: Scleraxis (*Scx*) for periodontal ligament cells; Keratocan (*Kera*) for osteoblasts; and Dentin matrix acidic phosphoprotein-1 (*Dmp1*) for osteocytes. The expression of each cell–specific marker gene was higher in the corresponding fraction, demonstrating that the fractionation was successful (Fig. 3b).

Based on this novel fractionation strategy, the source of RANKL was examined. Notably, osteocytes abundantly expressed RANKL, whereas the expression of RANKL in periodontal ligament cells and osteoblasts was scarce (Fig. 3b). These findings suggest that osteocytes are a major source of RANKL in periodontal tissue and that they have a potential ability to support osteoclastogenesis during orthodontic tooth movement.

### Osteocytes regulate orthodontic tooth movement *via* RANKL

**Figure 4:**
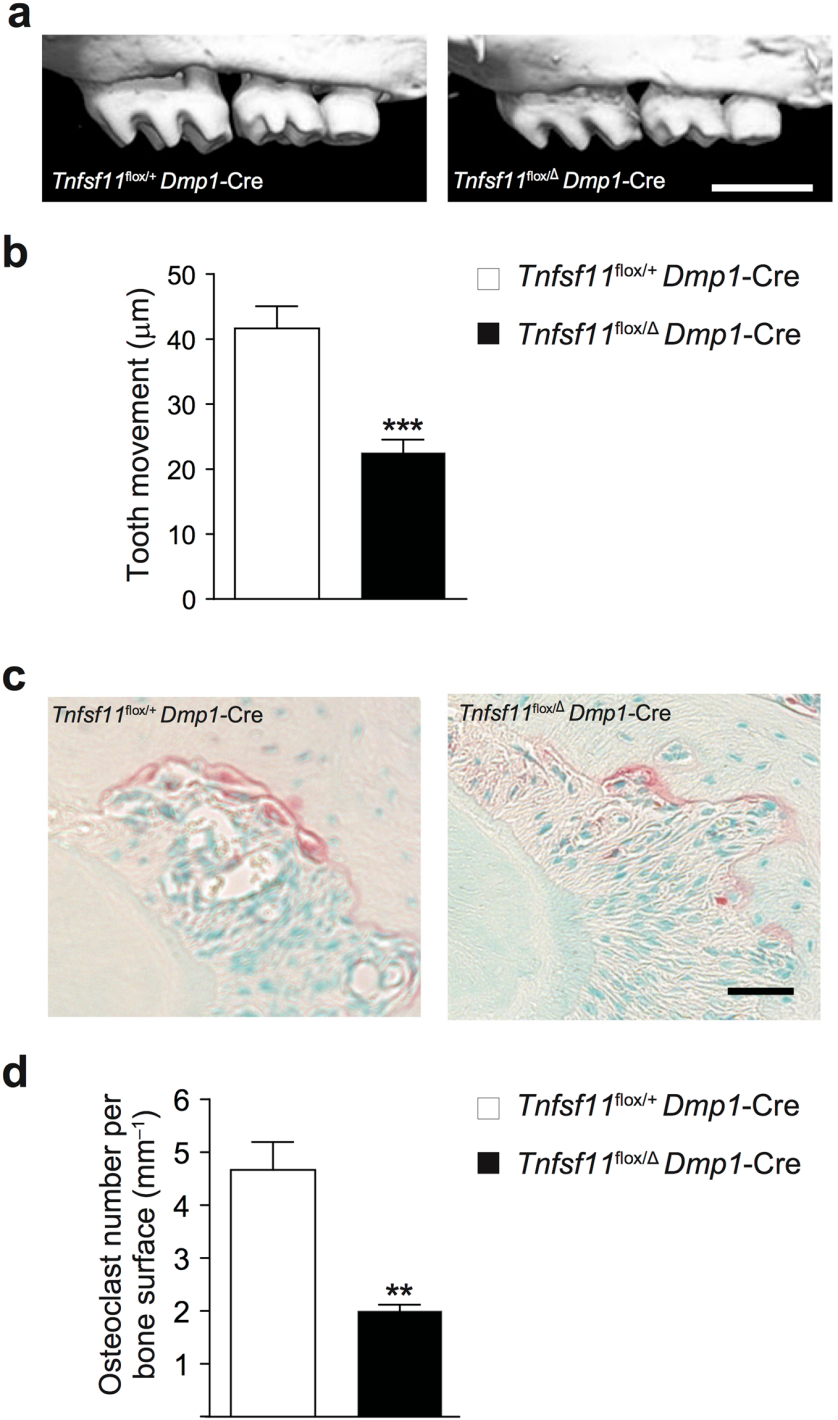
Tooth movement in osteocyte-specific RANKL deficient mice. (**a**) Representative micro-CT images of a moved tooth. *Tnfsf11* ^flox/+^ *Dmp1*-Cre mice were used as a control. (**b**) Quantification of tooth movement in each group (n = 6–7). (**c**) TRAP staining of the compression side of the palatal roots. (**d**) The number of osteoclasts on the alveolar bone surface. Scale bars: (**a**), 1 mm; (**c**), 50 μm. Error bars, means ± s.e.m.; **P < 0.01; ***P < 0.001.

We generated mice with osteocyte-specific deletion of RANKL by crossing mice carrying a *Tnfsf11* ^flox^ allele and mice expressing Cre recombinase under the control of *Dmp1* promoter (*Tnfsf11* ^flox/Δ^ *Dmp1*-Cre mice)^14^ to demonstrate the significance of osteocytes as a major source of RANKL *in vivo*. Orthodontic tooth movement was conducted in osteocyte-specific *Tnfsf11*-deficient mice. Micro-CT analysis clearly showed that orthodontic tooth movement was diminished in osteocyte-specific RANKL deficient mice compared with control littermate mice (*Tnfsf11* ^flox/+^ *Dmp1*-Cre mice) (Fig. 4a,b). Consistent with this, the number of osteoclasts on the compression side of the periodontal ligament space was dramatically reduced in osteocyte-specific *Tnfsf11* deficient mice **(**Fig. 4c,d). These results show that mice specifically deficient in *Tnfsf11* in osteocytes are resistant to orthodontic tooth movement due to the inhibition of osteoclastogenesis in periodontal tissue. Interestingly, the number of osteoblasts on the tension side of periodontal ligament space was also significantly decreased in the osteocyte-specific *Tnfsf11*–deficient mice (Supplementary Fig. 2), possibly through a coupling mechanism in alveolar bone. Thus, osteocyte-derived RANKL plays a crucial role in the regulation of osteoclastogenesis during orthodontic tooth movement.

## Discussion

The molecular mechanism of orthodontic force-induced tooth movement is poorly understood. Although previous studies have suggested that periodontal tissue component cells express RANKL and induce osteoclastgenesis^20, 21^, the source of RANKL in orthodontic tooth movement has not been elucidated because of the lack the cell-specific gene deletion system. This study shows that RANKL expressed by osteocytes functions as a potent driving factor in the induction of osteoclastogenesis during orthodontic tooth movement (Supplementary Fig. 3). Based on the results obtained from cell-specific gene deletion in mice, the RANKL derived from mesenchymal cells including chondrocytes and osteoblasts has a role in the regulation of osteoclastogenesis during bone modeling phase of skeletal development^15^. In contrast, osteocyte-derived RANKL contributes significantly to the physiological bone remodeling phase^14–16^.

Osteocytes constitute the majority of the cells in bone tissue and are thought to contribute to the regulation of bone remodeling in response to endocrine and mechanical stimuli^4, 5^. However, there has been limited evidence for osteocyte regulation of alveolar bone remodeling under orthodontic force^19^. Orthodontic force stimulates cells within the periodontal tissue, leading to cellular responses that result in alveolar bone remodeling. Tooth movement is associated with osteoclastic alveolar bone resorption on the compression side, which is the side that receives the compression force. We demonstrated that the inhibition of RANKL by a neutralizing antibody significantly reduced orthodontic tooth movement (Fig. 2). These results indicate that RANKL–RANK signaling is a key regulator of orthodontic force-induced osteoclastogenesis and tooth movement. This is further supported by previous reports: the inhibition of orthodontic tooth movement by local injection and gene transfer of osteoprotegerin (OPG encoded by *Tnfrsf11b*)^22, 23^, a decoy receptor of RANKL; the acceleration of orthodontic tooth movement by the local gene transfer of RANKL using a virus vector^24^; and the acceleration of orthodontic tooth movement in global *Tnfrsf11b*–deficient mice^25^. Neutralization antibody for human RANKL, Denosumab, has been used for osteoporosis and bone metastasis of tumors. Inhibition of RANKL–RANK signaling would be effective in anchorage control and retention, providing optimal orthodontic treatment outcome.

Identification of osteoclastogenesis-supporting cells during orthodontic tooth movement has long been an issue that needs to be addressed. Attempts have been made by *in situ* hybridization and immunohistochemical analyses, showing that RANKL is expressed by various cell types, including periodontal ligament cells, osteoblasts and epithelial cells^20, 21^. However, it has been unclear whether these cells are functionally relevant. In this study, we established a novel isolation method for obtaining each cell type from periodontal tissue (Fig. 3a). Using this method, we clearly showed osteocytes expressed a much higher amount of RANKL compared with periodontal ligament cells and osteoblasts (Fig. 3b).

Although osteocytes have been thought to be the functional regulator in orthodontic tooth movement^18, 26^, it is not well-understood how they contribute to the alveolar bone remodeling *via* orthodontic force. Orthodontic force-induced osteoclastogenesis and tooth movement were found to be greatly reduced in osteocyte-specific *Tnfsf11*-deficient mice (Fig. 4a,b). These findings clearly demonstrate that osteocytes are the major source of RANKL in periodontal tissue and choreographing alveolar bone remodeling under mechanical force (Supplementary Fig. 3). Because osteocytes are potential mechanosensory cells, it is suggested that osteocytes may express RANKL in response to mechanical force^14, 15^. Several possible mechanisms have been proposed on how mechanical force stimulates osteocytes and further studies will ultimately reveal the precise mechanism.

For the purposes of orthodontic treatment, not only the acceleration but also the suppression of tooth movement is necessary. The suppression is applied for the enforcement of anchorage, which prevents unwanted tooth movement. Many devices, including implants, are used for successful anchorage. However, these devices can damage periodontal tissues and the development of the drugs that modulate tooth movement is desired. To date, a various cytokines and compounds including prostaglandin E2, vitamin D3, calcitonin and bisphosphonates have been introduced as candidate drugs for optimizing tooth movement by regulating osteoclasts. Although these factors successfully modulated tooth movement in animals, they are not in clinical use due to potential side effects.

Our results suggest that local application of drugs that target osteocytes and regulate their RANKL expression would help anchorage management without damaging oral tissues. This study shed light on the regulation of the induction of osteoclasts in alveolar bone *via* orthodontic force, and may provide a molecular basis for novel therapeutic approaches to orthodontic treatment.

## Methods

### Experimental animals

Osteocyte-specific *Tnfsf11* deficient mice were generated by crossing *Tnfsf11* flox and *Dmp1*-Cre transgenic mice (*Tnfsf11* ^flox/Δ^ *Dmp1*-Cre mice), as previously described^14^. In all the experiments, littermates were used as control mice (*Tnfsf11* ^flox/+^ *Dmp1*-Cre mice). Wild-type mice (C57BL6/J) were purchased from CLEA Japan (Tokyo, Japan). Twelve-week-old male mice were used in all experiments unless otherwise described. All of the animal experiments were approved by the Institutional Animal Care and Use Committee and Genetically Modified Organisms Safety Committee of Tokyo Medical and Dental University (approval No. 0170317A and 2015-007C, respectively) and conducted in accordance with the guidelines concerning the management and handling of experimental animals.

### Experimental protocol for orthodontic tooth movement

Experimental tooth movement was conducted as described previously, with a modification^18, 27^. Briefly, after mice were anaesthetized, nickel-titanium closed-coil springs were inserted between the maxillary left first molar and the maxillary incisors with 0.9 mm stainless-steel ligature wires. Nickel-titanium coil springs provide mechanical force continuously.

### Maxillary bone resection and fixation

Eight days (unless otherwise indicated) after spring insertion, mice were killed. Maxillary bone was harvested by transecting the skull at the distal border of the orbit, followed by the removal of the nasal and the frontal bones. The obtained maxillary bone was fixed in 70% ethanol or 4% paraformaldehyde (PFA), for the evaluation of tooth movement or histological analyses, respectively^14, 28^.

### Measurement of orthodontic tooth movement

Micro-CT scanning was performed using a ScanXmate-A100S Scanner (Comscantechno)^14, 29^. Three-dimensional microstructural image data were reconstructed using TRI/3D-BON software (Ratoc System Engineering). Tooth movement was evaluated by measuring the closest distance between the first molar and the second molar in micro-CT images using Image J software (National Institutes of Health).

### Administration of anti-mouse RANKL monoclonal antibody (OYC1)

OYC1^30^ was purchased from Oriental Yeast (Tokyo, Japan). Purified Rat IgG2aκ (isotype control) was purchased from BioLegend (San Diego, US). Mice were injected daily for 8 days with 10 μg of the OYC1 or control IgG in 10 μL of phosphate-buffered saline (PBS) by using a microliter syringe (HAMILTON) with a 30 G needle (BD Biosciences). OYC1 or control IgG was administered into the buccal and palatal gingiva around the maxillary left first molar during the course of orthodontic tooth movement under general anesthesia.

### Histological analyses

After fixation, each sample was soaked in OSTEOSOFT (Merck Millipore) at 4 °C for 4 weeks in order to decalcify bone and tooth. The samples were embedded in paraffin, and 6-μm serial transverse sections of the first molar region were prepared. At least 5 serial sections were evaluated for each animal and for each analysis. TRAP and HE staining procedures were performed as previously described^14, 29^. For TRAP staining, methyl green was used for nuclear counterstaining.

### Bone histomorphometric analyses

The number of osteoclasts in the periodontal ligament space on the compression side of the distal-buccal root of the upper first molar was counted. Osteoclasts were identified as TRAP-positive multinucleated cells. The osteoblast surface on the tension side of the same root was measured. Histomorphometric parameters were calculated using Image J software.

### Isolation and gene expression profiling in periodontal tissue

After the maxillary bones were obtained, molars were extracted with a 28 G needle under microscopy. Soft tissues were removed and washed away in PBS with vibration. Then the maxillary bones were subjected to 3 times sequential digestion in a mixture containing 0.1% collagenase (Wako) and 0.2% dispase II (GODO SHUSEI) at 37 °C with shaking at 150 rpm for 15 min. After the first fraction was removed, the second fraction was collected as the osteoblast-rich fraction and resuspended in PBS. The remaining bones were used for the osteocyte-rich fraction. To obtain periodontal ligament cells, soft tissue around the extracted teeth was subjected to RNA extraction using ISOGEN (Wako).

### Quantitative RT-PCR analyses

Total RNA and cDNA were prepared by using ISOGEN and an RNeasy Micro Kit (QIAGEN) along with Superscript III reverse transcriptase (ThermoFisher Scientific) according to the manufacturers’ instructions^14, 29^. Real-time quantitative PCR analysis was performed with a LightCycler (Roche) using SYBR Green (TOYOBO). Gene expression values were calculated based on the ΔΔCt method using *Gapdh* expression as an internal control. The primer sequences were: *Dmp1*, 5′-CCCAGAGGGACAGGCAAATA-3′ and5′-TCCTCCCCACTGTCCTTCTT-3′; *Gapdh*, 5′-GGATGCAGGGATGATGTTCT-3′ and 5′-AACTTTGCCATTGTGGAAGG-3′; *Kera*, 5′-TCCCCCATCAACTTATTTTAGC-3′ and 5′-GGTTGCCATTACAGGACCTT-3′; *Scx*, 5′-ACACCCAGCCCAAACAGAT-3′ and 5′-TCTGTCACGGTCTTTGCTCA-3′; *Tnfsf11*, 5′-AGCCATTTGCACACCTCAC-3′ and 5′-CGTGGTACCAAGAGGACAGAGT-3′.

### Statistical analyses

All the data are representative of more than three independent experiments and were initially tested with F test or Bartlett’s test for normality distribution. If homoscedasticity could be assumed, they were parametrically analyzed using Student’s *t*-test or one-way analysis of variance (ANOVA) followed by Tukey’s multiple-comparison test. In a case where homoscedasticity could not be assumed (see figure legend), non-parametric Kruskal–Wallis test followed by Dunn’s multiple-comparison test was applied for multiple comparison. Differences with a P value of <0.05 were considered significant (*P < 0.05; **P < 0.01; ***P < 0.001; ****P < 0.0001; NS, not significant, throughout the paper). All data are presented as the mean ± standard errors of the mean values of independent replicates. All statistical analyses were performed with Prism Version 5 (GraphPad Software Inc.).

## Electronic supplementary material

Supplementary information
