# Supplementary material for: Osteocyte regulation of orthodontic force-mediated tooth movement via RANKL expression
Source: Sci Rep. 2017 Aug 18;7:8753. doi: 10.1038/s41598-017-09326-7 (PMC5562866; doi:10.1038/s41598-017-09326-7)
Supplement: Supplementary file 1 — Supplementary information [file 41598_2017_9326_MOESM1_ESM.pdf]

SUPPLEMENTARY INFORMATION

Osteocyte regulation of orthodontic force-mediated tooth movement *via* RANKL expression

Ayumi Shoji-Matsunaga[1,2], Takehito Ono[1], Mikihito Hayashi[1], Hiroshi Takayanagi[3], Keiji Moriyama[2], Tomoki Nakashima[1,4,5]

Correspondence and requests for materials should be addressed to T.N (email: naka.csi@tmd.ac.jp)

Supplementary figure legends

Supplementary Figure 1. Osteoblastic bone formation during orthodontic tooth movement. (**a**) Micro-CT images of sagittal sections of the maxillary bones in wild–type mice after spring insertion (0, 4, 8 and 12 days). (**b**) Osteoblast surface per alveolar bone surface (n = 3 per each time point). Scale bar: 1 mm. Error bars, means ± s.e.m.; ****P < 0.0001; NS, not significant.

Supplementary Figure 2. Osteoblastic bone formation in osteocyte-specific RANKL deficient mice. Osteoblast surface per alveolar bone surface in each group (n = 3–4). The dotted lines denote the osteoblast surface. Scale bar: 50 μm. Error bars, means ± s.e.m.; ****P < 0.0001.

Supplementary Figure 3. Schematic diagram of orthodontic tooth movement. Osteocyte-derived RANKL regulates osteoclastogenesis during orthodontic tooth movement.

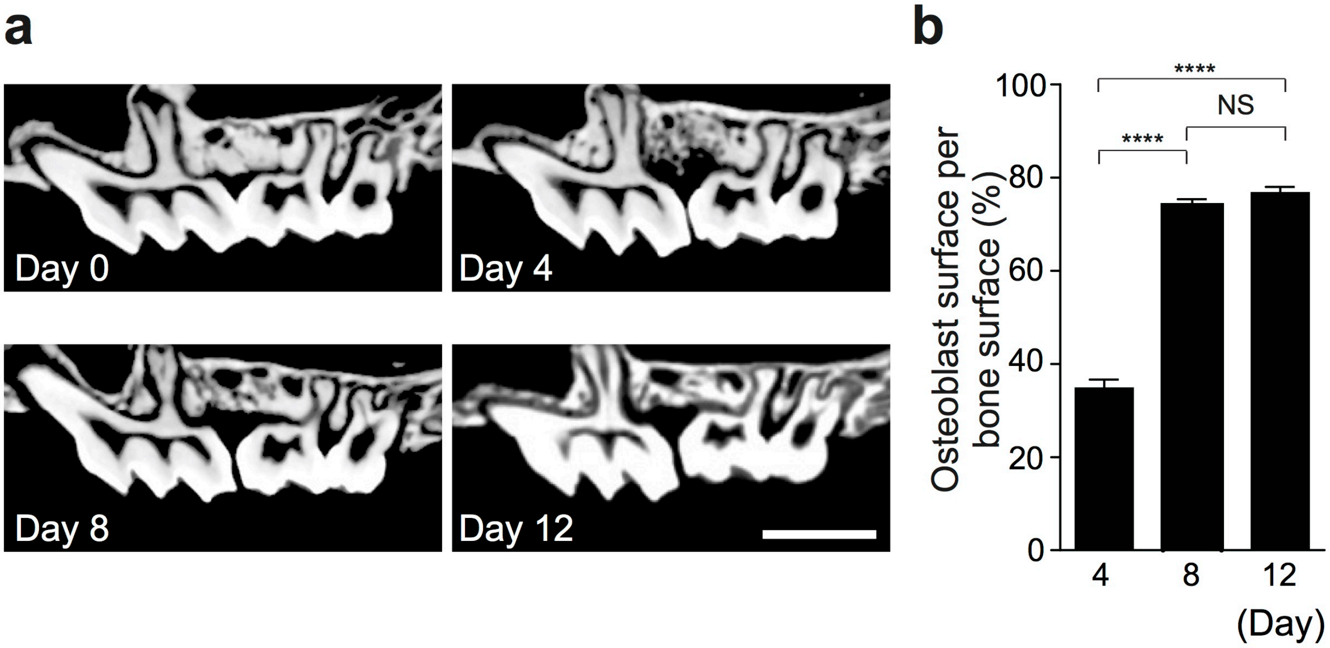
a
Day 0
Day 4
Day 8
Day 12
b
Osteoblast surface per bone surface (%)
0
20
40
60
80
100
****
NS
****
4
8
12
(Day)

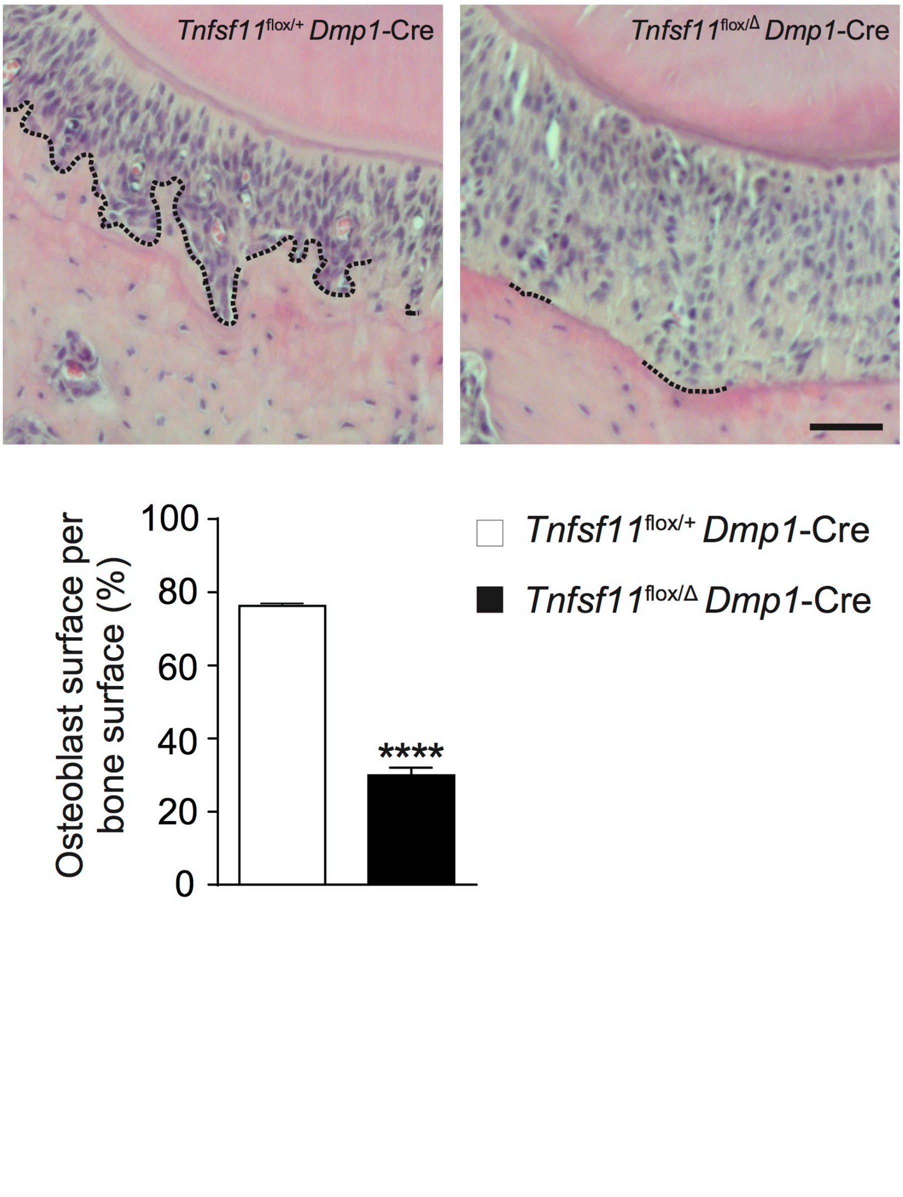

Tnfsf11$^{flox/+}$ *Dmp1*-Cre
Tnfsf11$^{flox/\Delta}$ *Dmp1*-Cre
Osteoblast surface per bone surface (%)
100
80
60
40
20
0
****
Tnfsf11$^{flox/+}$ *Dmp1*-Cre
Tnfsf11$^{flox/\Delta}$ *Dmp1*-Cre

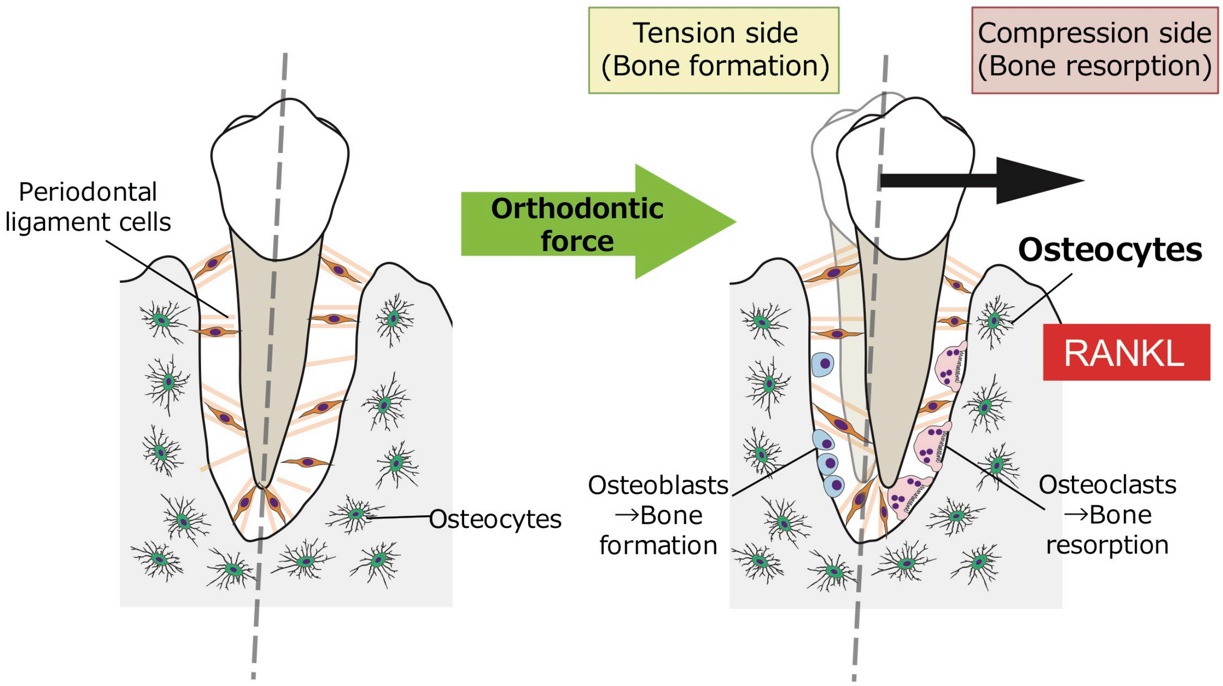
Periodontal
ligament cells
Osteocytes
Orthodontic
force
Tension side
(Bone formation)
Compression side
(Bone resorption)
Osteocytes
RANKL
Osteoblasts
→Bone
formation
Osteoclasts
→Bone
resorption
